# Supplementary figures and images for: Up-regulation of plasma miRNA-21 and miRNA-422a in postmenopausal osteoporosis
Source: PLoS One. 2023 Oct 18;18(10):e0287458. doi: 10.1371/journal.pone.0287458 (PMC10584188; doi:10.1371/journal.pone.0287458)

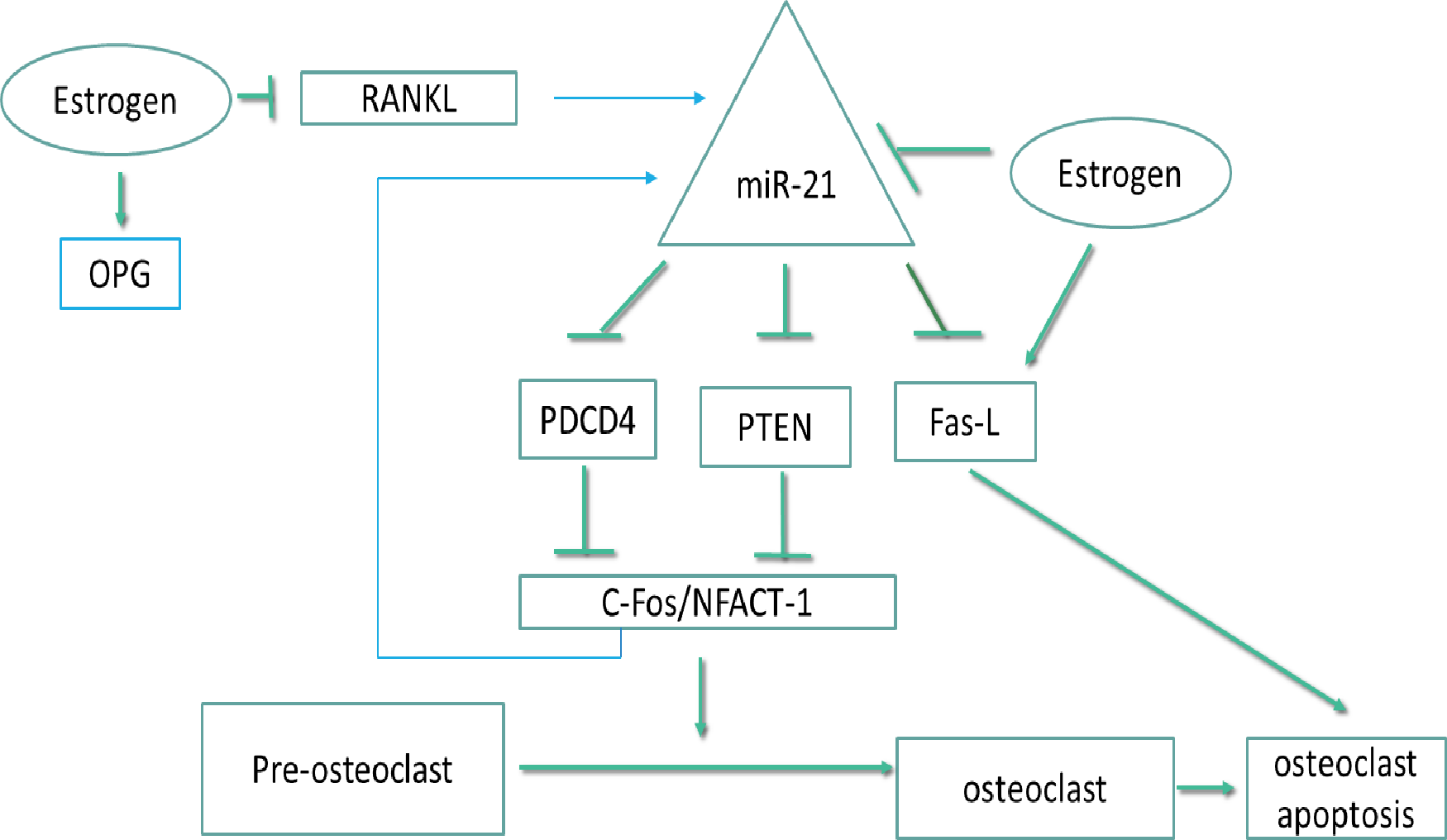

Supplement: S1 Fig — (TIF) [file pone.0287458.s001.tif]

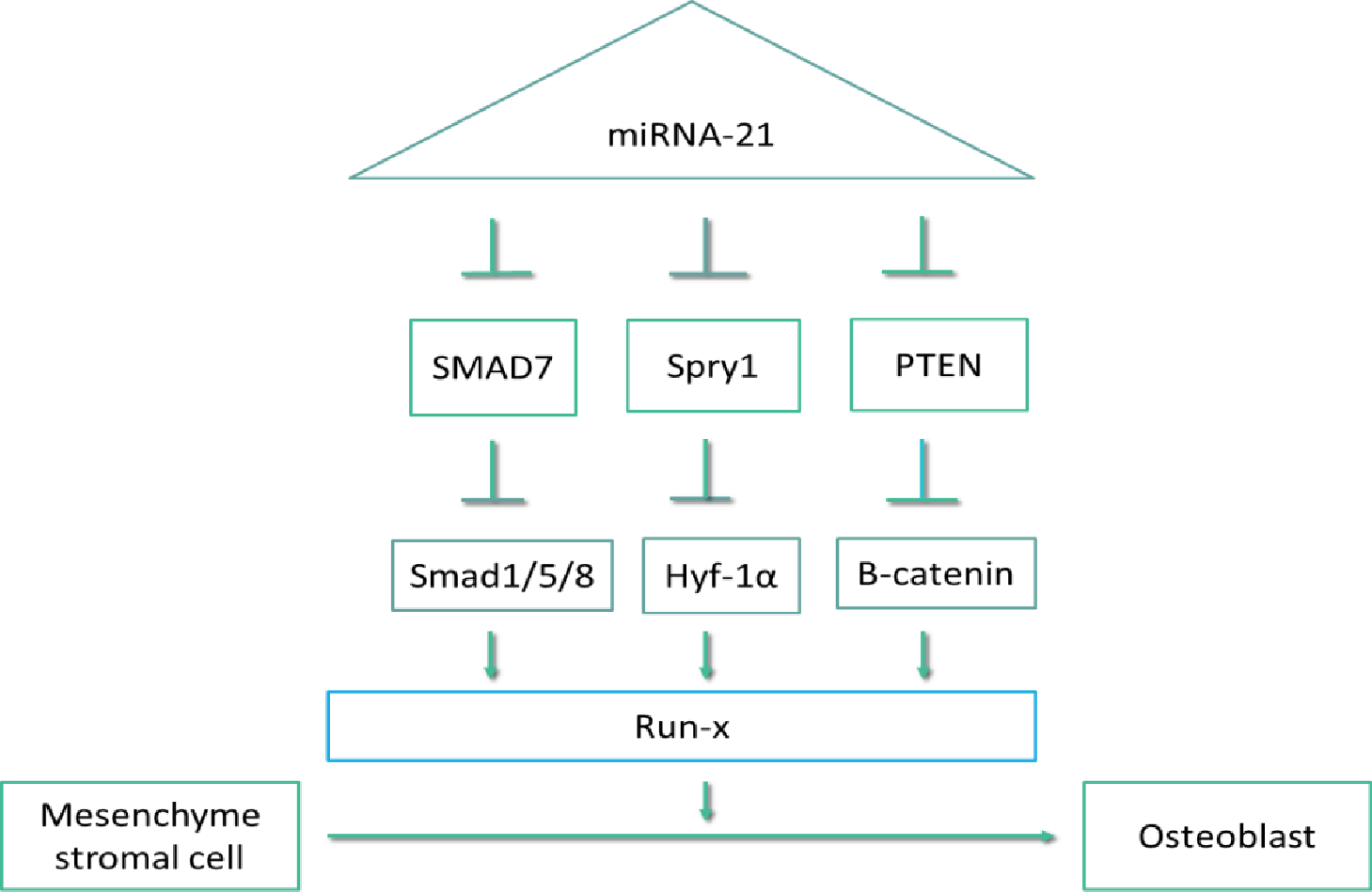

Supplement: S2 Fig — (TIF) [file pone.0287458.s002.tif]
